# A Comprehensive Review of Cholinesterase Modeling and Simulation

**DOI:** 10.3390/biom11040580

**Published:** 2021-04-15

**Authors:** Danna De Boer, Nguyet Nguyen, Jia Mao, Jessica Moore, Eric J. Sorin

**Affiliations:** 1 Department of Chemistry & Biochemistry, California State University, Long Beach, CA 90840, USA; Danna.DeBoer@student.csulb.edu; 2 Department of Chemical Engineering, California State University, Long Beach, CA 90840, USA; Nguyet.Nguyen01@student.csulb.edu (N.N.); Jia.Mao@student.csulb.edu (J.M.); 3 Department of Biomedical Engineering, California State University, Long Beach, CA 90840, USA; Jessica.Moore@student.csulb.edu

**Keywords:** acetylcholinesterase, butyrylcholinesterase, docking, molecular dynamics, hydrolysis, molecular recognition, catalysis, inhibition, reactivation

## Abstract

The present article reviews published efforts to study acetylcholinesterase and butyrylcholinesterase structure and function using computer-based modeling and simulation techniques. Structures and models of both enzymes from various organisms, including rays, mice, and humans, are discussed to highlight key structural similarities in the active site gorges of the two enzymes, such as flexibility, binding site location, and function, as well as differences, such as gorge volume and binding site residue composition. Catalytic studies are also described, with an emphasis on the mechanism of acetylcholine hydrolysis by each enzyme and novel mutants that increase catalytic efficiency. The inhibitory activities of myriad compounds have been computationally assessed, primarily through Monte Carlo-based docking calculations and molecular dynamics simulations. Pharmaceutical compounds examined herein include FDA-approved therapeutics and their derivatives, as well as several other prescription drug derivatives. Cholinesterase interactions with both narcotics and organophosphate compounds are discussed, with the latter focusing primarily on molecular recognition studies of potential therapeutic value and on improving our understanding of the reactivation of cholinesterases that are bound to toxins. This review also explores the inhibitory properties of several other organic and biological moieties, as well as advancements in virtual screening methodologies with respect to these enzymes.

## 1. Introduction

**Figure 1:**
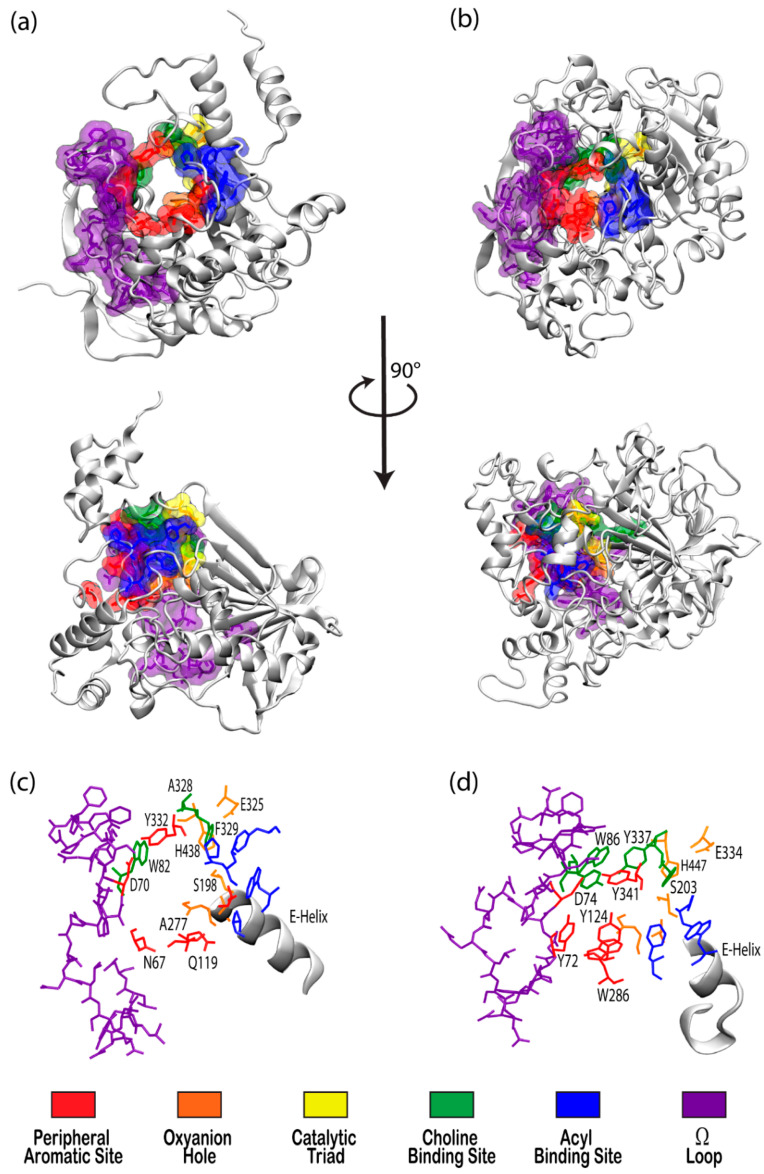
Visualizations of (**a**) butyrylcholinesterase (BChE) (PDBID 1P0I) and (**b**) acetylcholinesterase (AChE) (PDBID 1B41) in white ribbon mode with residues in notable binding sites shown as semi-transparent van der Waals surfaces colored according to the key. Top panels present views directly into the active site gorges with structures in the center panel rotated 90° about the vertical axis. Bottom panels present magnified views of the binding site regions of (**c**) BChE and (**d**) AChE with key residues labeled.

The cholinesterase enzyme family has but two members: acetylcholinesterase (AChE) and butyrylcholinesterase (BChE). The former’s primary biological purpose is regulating acetylcholine, a neurotransmitter, via hydrolysis at neuromuscular junctions, thus proving itself to be an essential component in the maintenance and performance of nervous systems. AChE, which has also been referred to as “true cholinesterase”, is created in muscle, nerve, and hematopoietic cells and is considered to be one of the most efficient enzymes due to its rapid rate of catalysis [1]. AChE’s plasma analog, BChE, previously referred to as “pseudocholinesterase”, is produced in the liver and, unlike AChE, has been viewed as having a much more ambiguous biological purpose, as it was long believed to be vestigial [2]. BChE has a higher concentration in plasma than AChE, is present in many vertebrates, and tolerates several mutations, which has prompted the theory that BChE evolved from AChE to be a general detoxifier while still retaining some function in the process of neurotransmission [3]. This theory seems apt given the structural similarity of their binding sites, their shared affinities for certain substrates and ligands, and their sequence homology of approximately 65% [4]. Figure 1 depicts both AChE (bottom) and BChE (top) from a gorge-centric view and after a 90° rotation.

One difference between the two enzymes is the relative size of the binding pocket, with BChE and AChE having approximate gorge volumes of 1500 Å^3^ and 1300 Å^3^, respectively [5]. X-ray crystallography of *Torpedo californica* (*Tc,* pacific ray) AChE revealed the enzyme to have a deep hydrophobic gorge with residues that stabilize substrates in the pocket [6], as well as a bottleneck region in the active site [7] that narrows to approximately 4 Å in width [8]. Common models of AChE, including human, mouse, and *Torpedo californica* (*Tc*AChE), demonstrate conserved active sites, save for a few residues that participate in ligand binding [9], and both have negative surface potentials that become more negative deeper within the gorge. This negative potential, which is high near the catalytic site at the “bottom” of the gorge, seems to have evolved to facilitate electrostatic attraction of positively charged choline substrates by both enzymes [8].

Although BChE is structurally similar to AChE, 6 of the 14 aromatic amino acids that line the active site gorge in AChE are substituted with aliphatic residues in BChE [10]. In particular, the substitution of Phe288 and Phe290 in *Tc*AChE with the smaller Leu286 and Val288 in human BChE lead to conformational changes that result in a deeper gorge in BChE, thereby allowing BChE to interact with, and potentially hydrolize, a much wider range of substrates and inhibitors than AChE [11]. BChE is thus characterized as the promiscuous, or non-specific, bigger sibling to the smaller and much more specific AChE.

The tremendous growth and improvement in computational resources and modeling techniques over the past few decades have led to an exponential increase in computational studies of biomolecular systems. In 2003, the Protein Data Bank (PDB) launched its online presence, thereby making myriad biomolecules and complexes available for structural and computational research, and by 2016 there were 178 AChE structures available to the public, many of those including bound substrates or inhibitors of medical and pharmacological significance [9]. In fact, some of the earliest and most significant contributions of cholinesterase models were reported by Sussman et al., who performed an X-ray analysis of AChE at 2.8 resolution in 1991 [12], and Nicolet et al., who examined several crystal structures of BChE in 2003 [13]. Modeling and simulation-based studies of the cholinesterase enzymes have advanced in tandem with this growth.

**Figure 2:**
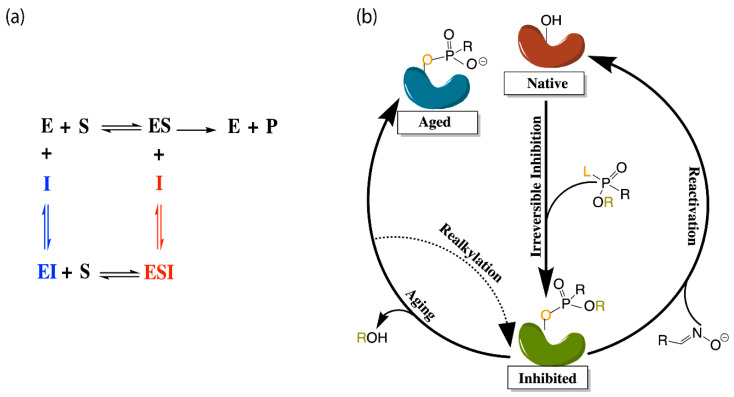
(**a**) Depiction of the enzyme catalytic mechanism (black) and reversible inhibition mechanisms including competitive inhibition (blue), uncompetitive inhibition (red), and noncompetitive inhibition (both blue and red). (**b**) Representation of irreversible (covalent) inhibition by organophosphorus ligands and the reactivation mechanisms to revert the aged enzyme (blue) back to the inhibited complex (green). L and R represent the first and second leaving groups of the organophosphate, respectively, following the paths of inhibition and reactivation.

The application of molecular dynamics (MD) simulations, Monte Carlo (MC) based docking calculations, and more sophisticated quantum mechanical/molecular mechanics (QM/MM) simulations have proven highly insightful in probing cholinesterase structure and activity. Notable areas of interest include the mechanisms of cholinesterase catalysis, reversible and irreversible inhibition of both enzymes to manage Alzheimer’s Disease (AD) and other ailments, BChE-specific inhibition, and the reactivation of phosphorylated cholinesterases, a process that normally follows nerve agent attacks or pesticide poisoning [14]. Figure 2a,b depict the common paths of substrate or ligand binding and phosphorylation, respectively.

Most therapeutic treatments of AD, and other illnesses to which the cholinesterases have been linked, are reversible inhibitors that form non-covalent molecular recognition (MR) complexes with cholinesterases and can leave the active site, thus existing in an equilibrium between bound and unbound states characterized by K_I_ and/or IC_50_ values. The three mechanisms that reversible inhibition can follow are competitive, noncompetitive, and uncompetitive, as depicted in Figure 2a. During competitive inhibition, the substrate and inhibitor are competing for the same binding site, and the binding of a competitive inhibitor within the active site blocks entrance of the substrate, thereby hindering formation of the enzyme substrate complex. By contrast, uncompetitive inhibitors function by binding to the enzyme-substrate complex to prevent product formation, and noncompetitive inhibitors can bind to either the enzyme or the enzyme-substrate complex to regulate catalytic activity.

Many organic compounds, including organophosphates (OPs), are categorized as irreversible inhibitors, which covalently bond to residues in the gorge and thus cannot leave the active site. In the cholinesterases, OPs covalently bind to the serine residue of the catalytic triad. Irreversibly inhibited cholinesterases, however, can be reactivated via various pathways with compounds such as oximes, as illustrated in Figure 2b. Oximes reactivate an inhibited cholinesterase to its native structure by nucleophilic substitution of the phosphorylated serine. If untreated, the inhibited enzyme can undergo the dealkylation process, also known as aging, where the loss of the second leaving group produces an oxyanion on the phosphoryl group.

Aged cholinesterase is highly stable due to the strong electrostatic interactions between the oxyanion and the positively charged catalytic histidine. Known reactivators of OP-inhibited AChE, such as oximes, are ineffectual against aged AChE [15]. Though a successful attempt to reactivate aged AChE by a class of compounds called “quinone methide precursors” (QMPs) was reported by Hadad and coworkers [16], the reaction between QMPs and aged AChE was rendered too slow to be useful [17]. The present review will examine many studies generally focusing on these areas of interest and, unless otherwise specified, all amino acid numbering used below will refer to human cholinesterase models.

## 2. Structure and Dynamics

While understanding the structure, function, and behavior of cholinesterase binding sites seems a practical starting point for modeling studies, early researchers initially wanted to understand how substrates and inhibitors entered the active sites of these enzymes. Molecular dynamics (MD) simulations of human BChE lasting for 5 and 10 ns indicated that inhibitors can access the binding subsites in the catalytic cavity due to the highly flexible entrance (or “mouth”) of the gorge, a portion of which is formed by the flexible omega loop (Ω-loop) region, as well as the peripheral aromatic site (PAS, formerly mistakenly called the peripheral anionic site). In these simulations, the Asp70 residue in PAS showed significant deviation from the crystal structure with root-mean-square deviation (RMSD) values of 2 to 6 Å [18]. AChE has also been shown to experience such fluctuations at the gorge entrance, with a similarly flexible Ω-loop region that is thought to increase enzyme specificity by making it more difficult for large molecules to enter the AChE gorge without hindering the productivity of the enzyme [19].

Apart from the fluctuations at the mouth of the gorge, AChE also experiences what is known as bottleneck fluctuations, with the bottleneck being the narrowest part of the gorge. These fluctuations, which have come to be known as the “breathing” of the enzyme, can help inhibitors or substrates move from the surface to deeper regions of the gorge [20]. Cheng et al. recently defined this breathing by monitoring the varying distance between the C_ε2_ atom of Phe330 (CBS) and the O_H_ atom of Tyr121 (PAS) in TcAChE, which suggested that a number of subdomains within the enzyme, particularly the Ω-loop, contribute to modulating the size of the gorge bottleneck [21]. A comprehensive comparison between 47 crystal structures of AChE (in its native form and in complex with small molecules), as well as a 20 ns simulation of *Tc*AChE, provided by Sussman and coworkers, suggested that the 14 aromatic residues lining the AChE gorge and creating over half of the gorge surface area contribute greatly to the overall flexibility of the enzyme, the observed bottleneck breathing motions, and the resulting ability to perform its catalytic function [22]. 

Some of these aromatic residues play significant roles in primary subsites within the cholinesterase gorges, such as the catalytic active site (CAS) and the peripheral aromatic site (PAS) [22]. While these sites, which are addressed in more detail below, are integral to cholinergic activity, cholinesterases do not only perform cholinergic functions. As discussed by Chinnadurai et al., the aryl acylamidase activity (AAA) of AChE, which also involves hydrolysis, only requires the CAS and does not interact with the PAS at all [23]. This has prompted the theory that AAA substrates enter from a side-door into the enzyme, rather than via the mouth of the gorge and, indeed, researchers have suggested that there are a number of doors through which substrates can enter the gorge including a back door [24], an Ω-loop door, and the suggested side door [23]. It was suggested that the side door may open more frequently than the other doors to mediate AAA activity, and MD simulations of side door probing emphasize the importance of hydrophobic interactions, hydrogen bonding, and water mediated interactions (“water bridges”) in moving the substrate towards the CAS [23]. To be sure, simulations of AChE in explicit solvent sans substrate [25], as well as analyses of *Tc*AChE crystal structures in its native and several inhibited forms [26], have underscored the importance of the presence of molecular water in enzyme structure.

At higher concentrations, dimerization and the further dimerization of dimers to form tetramers is known to affect the structure and function of cholinesterase enzymes [27]. While MD simulations suggested that two of the four binding sites in tetramerized cholinesterases are sterically blocked, thereby becoming less active [28], as reflected by a 15% decrease in catalytic activity [29], a recently elucidated CryoEM structure of hBChE shows distinct structural variance from the simulated tetramer, with the active site gorges being fully solvent accessible [27]. Tetramerization, however, increases the half-life of the enzymes, which is a desirable result when cholinesterases, and BChE in particular, are being used to counteract drug overdoses. For example, the addition of proline-rich attachment domains (PRADs) to BChE increases tetramer stability, leading to an extended circulation time [30]. Interestingly, glycosylated models of BChE increase the enzyme’s flexibility and half-life without hindering its ability to bind to glycans, which cannot be said of all therapeutic protein targets [31]. On the other hand, cholinesterase phosphonylation, or the irreversible binding of organophosphate to the active site, which will be discussed in more detail in the organophosphate inhibition section below, severely restricts the flexibility of both AChE and BChE, as reported by Bennion et al. [32]. Experimental findings of AChE covalently bound to the nerve agent soman agree that OP-poisoned AChE is significantly stiffer [33].

### 2.1. Important Binding Sites

There has been substantial past effort to study the sites responsible for molecular recognition (MR) and binding affinity within the active site gorge of both enzymes, and it is clear that those binding sites, specific chemical subsites within each active site gorge, mirror each other and perform similar functions respective to each enzyme. While it is important to note that these binding sites have been examined experimentally with X-ray and kinetic studies, including a recent study by Rosenberry et al. [34], the present review focuses on the unique perspective provided by computational investigations. As expected, one of the most important sites for both cholinesterases is the catalytic active site (CAS), and the peripheral aromatic site (PAS) also plays an indispensable role in cholinesterase or ligand binding, while the Ω-loop (OML), acyl binding site (ABS), and oxyanion hole (OAH) sites are more essential in contributing to binding affinity and complex stability. Alvarado et al. have provided a method of succinct graphical tabulation of BChE-ligand contacts and interactions, referred to as contact tables, that include these five sites and additional protein residues of interest [35], as discussed below. A detailed analysis of these binding sites is provided here in the same order that they are encountered by substrates and inhibitors upon entering and moving into the gorge.

#### 2.1.1. Peripheral Aromatic Site

The peripheral aromatic site (PAS, red in Figure 1) is located near the mouth of gorge [36] and plays a prominent role in substrate and ligand binding [37]. For decades, peer-reviewed studies have used PAS to denote the “peripheral anionic site”. In recent years, however, the aromatic properties of this binding site that are vital to cholinesterase function have driven the community to instead refer to this region as the “peripheral aromatic site”. Important amino acids in the PAS of AChE include serine, tyrosine, aspartic acid, and tryptophan [36,38], while notable PAS residues in BChE include asparagine, aspartic acid, glutamine, serine, and tyrosine [35], highlighting the polar, negatively charged, and electron-rich nature of residues in this site. As previously mentioned, one distinction between the cholinesterases is the aromatic nature of the residues surrounding the PAS in AChE, which is more aliphatic in BChE [10]. The PAS makes contact with many loops and secondary structural elements at the surface of the protein, including the Ω-loop, which contributes to the needed flexibility discussed above. Although steric and electrostatic interactions may slow the catalytic efficiency of AChE, the PAS is valuable for trafficking ligands into the gorge [39], particularly positively charged species such as cholines. MD simulations have emphasized the importance of cation-π interactions, which stabilize the ligand at the rim of the gorge entrance prior to entering the gorge [40], and it has been proposed that non-cholinergic activity of the PAS could include the deposition of amyloids, adhesion to cells, and outgrowth of neurites [41].

#### 2.1.2. Acyl and Choline Binding Sites

Once a ligand has entered the gorge, the acyl and choline binding sites (ABS and CBS, shown as blue and green in Figure 1, respectively), which are located near the catalytic triad, assist in positioning the ligand for catalysis. The ABS and CBS are hydrophobic regions composed primarily of tryptophan, tyrosine, and phenylalanine in human AChE. Tyr337 in the choline binding site of AChE is replaced by Ala328 in that of BChE; Phe295 and Phe297 in the acyl binding site of AChE are replaced by Leu286 and Val288, respectively, in BChE. The replacement of aromatic residues in the ABS and CBS of BChE enable it to bind larger substrates and inhibitors than AChE [42]. In addition, the ABS and CBS are largely responsible for the specificity of these enzymes and are thus primary targets studied when synthesizing inhibitors such as imidazole or pyridine derivatives [43].

#### 2.1.3. Catalytic Active Site

The catalytic active site (CAS, yellow in Figure 1) is located approximately 20 Å deep at the bottom of both the AChE and BChE gorges [12,24] and is made up of serine, glutamic acid, and histidine residues, prompting the name “catalytic triad” [24,35]. The CAS is surrounded by numerous aromatic and acidic residues [44] and is observed to engage in shorter hydrogen bonds in crystal and NMR structures than observed in simulation [45,46]. More importantly, the CAS is highly conserved [44], emphasizing historically vital biological roles of these enzymes and their cholinergic activities. QM/MM simulations at the MP2(6-31 + G*) level reveal a potential energy barrier of 10.5 kcal/mol, which agrees with experimental data [47], and MD simulations of AChE bound to acetylcholine (ACh) show that ACh stabilizes the CAS and improves the binding ability of the peripheral aromatic site [48].

It has been postulated that a back-door exists in AChE, just behind the CAS and controlled by a tryptophan residue, which was theorized after a single water molecule exited the active site gorge from a direction opposite that of the gorge entrance in an MD simulation [49]. This “back door” was later thought to open three to four Å wide such that catalysis products could exit the gorge of the enzyme without blocking the gorge entrance, and thus contributing to a high catalytic rate [50]. Aromatic residues surrounding the CAS histidine also largely influence the productivity and efficiency of the enzyme [51], which decreased approximately 600-fold when disrupted or replaced by aliphatic side chains [52]. More recently, Xu et al. used MD to study *Tc*AChE and, from 27 of their 40 trajectories, observed thiocholine to frequently exit the active site via a back-door created by cooperative motions of CAS residue Trp86 along with Val132 and Gly448 [53], for which previous experimental support was noted [54,55].

Indeed, other mutations in or near the CAS are known to have effects on the structure, and subsequent function, of the enzyme. This research has naturally focused on, and is more applicable to, BChE due to its much greater natural affinity for mutations than AChE [3]. For example, prolonged use of muscle relaxers led to the discovery of the “silent phenotype” in which an alanine is mutated to a valine near the CAS of BChE. This mutation was studied in silico and observed to severely disrupt interactions between the histidine and serine in the catalytic triad [56], leading to a dysfunctional CAS, regardless of the inhibitor, and increases in the volume of the enzyme, indicating that this may be a pre-denaturation state [57].

The mutation of a nearby alanine in silico to cystine in wild-type (WT) BChE causes the histidine in the catalytic triad to flip, an event that is largely guided by local water molecules [58]. This man-made mutation, while possibly slowing the speed of binding, ultimately still allows for substrate binding to the active site; the naturally occurring mutation of that alanine to aspartic acid, however, is claimed to be catalytically inactive due to strong disruptive interactions between aspartic acid and the CAS histidine residue [59]. Both mutations showcase the possible hysteretic behavior of BChE, or its reliance on past-states, which can likely be attributed to its toxicological and pharmaceutical functions [58,59]. For instance, a man-made mutant of BChE was recently modeled and examined by Masson and coworkers, using QM/MM and Markov state analysis, and was determined to be a template for future investigations into organophosphate hydrolase functions [60].

#### 2.1.4. Oxyanion Hole

The oxyanion hole (OAH, orange in Figure 1), made of two glycines and one alanine [35,47], is generally a two-pronged site in many proteases and hydrolases; in the case of AChE, and subsequently BChE, the OAH is a three-pronged hole [61]. Early MD simulations of AChE phosphonylation suggested that the OAH exerts a pulling force on leaving groups during the alkylation step [45], and the OAH is known to lower the energy barrier for ACh hydrolysis in both cholinesterases [62]. QM/MM simulations exhibited consistent, tight hydrogen bonding between the OAH and the carbonyl carbon of the substrate, suggesting that the OAH facilitates stabilizing interactions in intermediate and transition states [61].

#### 2.1.5. Ω-loop (Omega Loop)

The Ω-loop (OML, purple in Figure 1), consisting of a series of nearly 30 residues, is located along one side of the active site gorge wall. In the presence of a substrate or inhibitor, the Ω-loop plays an important role in modulating enzyme “breathing”, regulating the size of the gorge, and thereby enabling the passage of the ligand to the active site [21]. The OML undergoes conformational changes, such as gorge enlargement, through torsional motion and segmental fluctuations [63,64]. Unregulated motions and decreased electrostatic interactions, however, can significantly decrease the binding affinity of these enzymes, such as the case of the atypical mutation from Asp70 to Gly70 in the OML of BChE, which Masson et al. reported could increase the *K*_m_ values 10- to 100-fold [65]. Moreover, the Ω-loop is speculated to facilitate an alternative entrance, the proposed side-door model noted above, to the active site of the enzyme; MD simulations performed by Wiesner et al. suggested that protonation of the AChE active site leads to conformational changes within the Ω-loop at Asn87 and Glu84 that result in the opening of this side door [66]. A similar observation was recently reported by the Rydzewski laboratory for *Tc*AChE, where opening and closing of this side door due to the displacement of the OML favored alternative dissociate routes of the substrate and inhibitor [67]. To put these computational results into perspective, a number of experimental investigations into the omega loop and backdoor of AChE from various species have suggested that the back door opens in some cases [50,68,69], but also that this opening is likely not relevant functionally [70].

## 3. Catalysis

The primary catalytic function performed by AChE is hydrolysis of the neurotransmitter acetylcholine (ACh) [1], and molecular modeling and simulation studies have provided insight into this process that cannot be easily gleaned from experimental efforts. For example, MD simulations using an ab initio QM/MM potential were conducted to map the reaction mechanism of AChE with ACh, pointing to a mechanism with two main processes: acylation and deacylation. In the first step of acylation, the system must overcome the initial free energy barrier of 12.4 kcal/mol, during which the oxygen atom of Ser203 performs a nucleophilic attack at the carbonyl carbon of acetylcholine, with a synchronous proton transfer from Ser203 to His447, resulting in the first tetrahedral intermediate [71]. This intermediate is stabilized by local hydrogen bonds from the oxyanion region and electrostatic interactions with the glutamic acid in the catalytic triad [47]. The second step in acylation has an additional free energy barrier of 1.9 kcal/mol and is characterized by the proton transferring to the leaving group of ACh, resulting in bond cleavage and choline release from the pocket [71].

The next step in this process, deacylation, also occurs in two steps, the first of which includes interaction between a water molecule and the carbonyl carbon of the acetyl group in acetylcholine, leading to a second tetrahedral intermediate that is stabilized by the Gly121, Gly122, and Ala204 residues of the oxyanion hole. The proton then transfers to the acetylserine oxygen atom from His447, yielding the products acetic acid and AChE, with the initial free energy barrier of deacylation higher than that of acylation at 17.5 kcal/mol and thus predicted to be the rate-limiting step [71].

**Figure 3:**
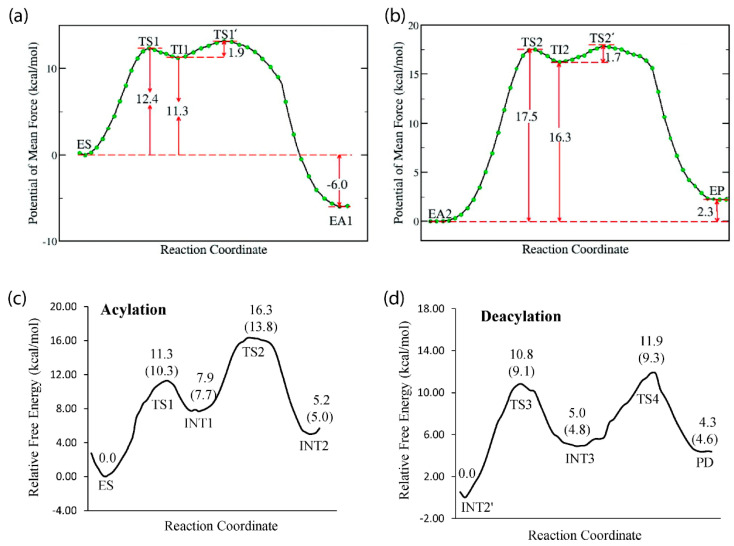
(**Top**) Free energy profiles for hydrolysis of ACh by AChE including the (**a**) acylation and (**b**) deacylation steps [71]. (**Bottom**) Free energy profiles for hydrolysis of ACh by BChE showing analogous (**c**) acylation and (**d**) deacylation steps [72].

Modeling efforts by Chen et al. have shown BChE to have a similar reaction pathway for hydrolysis of ACh and acetylthiocholine, with two-step acylation and deacylation processes [72,73]. Like AChE, residues in the oxyanion hole of BChE are integral for stabilization of the intermediate and catalytic function [73]. However, the acylation and deacylation processes for BChE were predicted to have free energy barriers of 13.8 and 11.9 kcal/mol, respectively, indicating that the rate-limiting step for BChE is not the deacylation step, as predicted for AChE, but rather the acylation step [72]. Figure 3 displays free energy profiles for the hydrolysis of ACh by both AChE and BChE to allow for a side-by-side comparison [71,72] of these results.

Given the promiscuous nature of BChE and its circulation in plasma, this enzyme has a much broader natural variety of substrates than AChE [2,3]. For example, BChE is one of the primary enzymes to hydrolyze heroin and produce its most active form, 6-monoacetylmorphine. Computational efforts by Zhan and coworkers showed this process to follow the two-step acylation and deacylation scheme outlined above [74]. In contrast, work by the same group to model BChE hydrolysis of ghrelin, the hunger hormone, showed a single-step acylation process [75]. Interestingly, Suarez et al. modeled BChE hydrolysis of butyrylcholine, a synthetic molecule that mimics acetylcholine and for which BChE is named [3], and found that the presence of glycerol or another butyrylcholine in the active site pocket stabilizes the intermediate product after transition state 2 in the deacylation step, which is a complex of BChE with butyric acid [76].

Cocaine is another substrate of interest for BChE catalysis studies, as increasing the catalytic efficiency of BChE for cocaine hydrolysis can be an effective method to treat overdoses, making BChE mutants and transition states important focal points for study. Rate-determining steps can be made faster with residue mutations [77] by changing local interactions and increasing substrate stability [78]. For example, computational mutations of non-active residues in the BChE active site gorge were predicted to increase catalytical efficiency as much as 1000-fold by strengthening hydrogen bonds [61]. The Ala199Ser/Ser287Gly/Ala328Trp/Tyr332Gly BChE mutant achieves this by increasing the strength of hydrogen bonds in the first transition state of BChE-cocaine catalysis and lowering the energy barrier [79].

Free energy perturbation (FEP) simulations have allowed researchers to see the deviation in free energy barriers at transition states for different BChE mutants and led to the discovery of a mutant, Ala328Trp/Ala199Ser/Phe227Ala/Glu441Asp/Ser287Gly, that is around 1800-fold more efficient than wild-type BChE [80]. Further work by Zhan and coworkers using FEP simulations showed the Ala328Trp/Tyr332Gly/Ala199Ser BChE mutant to have the potential to greatly increase catalytic efficiency and to thus serve as a potential means for exogenous therapy [81]. Using QM/MM simulations, Zhan’s team revealed two transition states for BChE-cocaine binding involving deformation of the non-prereactive complex and formation of the prereactive complex [77,82]. Indeed, efforts by the Zhan laboratory have resulted in a prodigious quantity and breadth of computational studies of cholinesterase catalysis in the 21st century.

## 4. Inhibition

We now turn to the area around which the majority of cholinesterase research has been dedicated over the past two decades: inhibition of one or both enzymes by various compounds, presented below in distinct chemical groupings including pharmaceuticals, narcotics, organophosphates, other organic species, and biological agents and salts. FDA-approved pharmaceutical inhibitors and their derivatives generally serve to treat the symptoms of AD. Due to the difference in size of the two enzymes, many drugs experience a wide range of selectivity and target multiple binding sites. As previously mentioned, the biological roles that BChE plays are much more ambiguous than those of AChE. However, hydrolysis of narcotics, such as nicotine and cocaine, has been identified as a possible role of BChE and, as noted above, this enzyme has been a target to treat narcotics overdoses.

**Figure 4:**
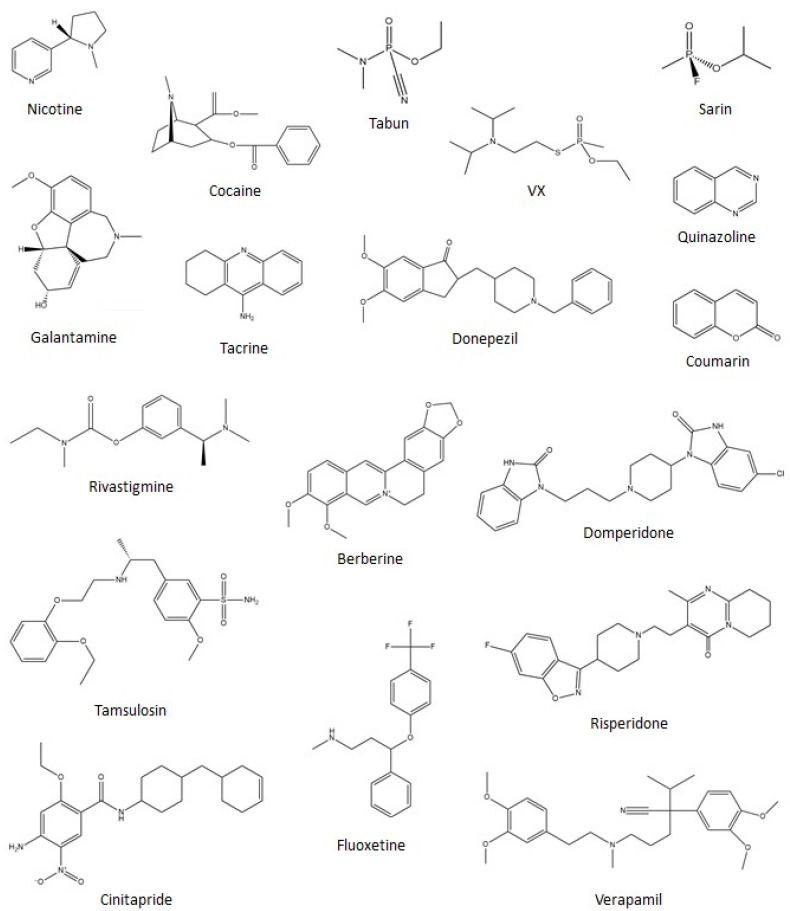
Chemical structures of various narcotic, nerve agent, and pharmaceutical cholinesterase inhibitors discussed in this review.

Organophosphate inhibitors have proven to have a wide field of study with myriad applications, and many phosphate-based molecules serve as potential cholinesterase inhibitors for disease management, while also being known for their highly toxic roles as irreversible inhibitors in nerve agents and pesticides. Finally, there are myriad compounds being researched, many aimed specifically at AD and other human ailments, that are still in early phases of research and not yet FDA-approved. Due to the vast number of unique inhibitors studied over the past two decades, many have been classified below under the broad category of other organic species, which are further organized according to prominent functional groups. Prior to this current effort, Anand and Singh reviewed different classes of cholinesterase inhibitors including tacrine, donepezil, rivastigmine, galantamine, xanthostigmine, para-amino-benzoic acid, coumarin, flavonoid, and pyrrolo-isoxazole analogues in their 2013 article [83]. Figure 4 presents molecular structures for many of the pharmaceuticals, narcotics, nerve agents, and related inhibitors mentioned above and detailed in the sections below.

### 4.1. Pharmaceuticals

#### 4.1.1. Tacrine and Derivatives

Tacrine, or 1,2,3,4-tetrahydroacridin-9-amine, was a commonly-used drug to treat AD (under the brand name Cognex) that was the first FDA-approved cholinesterase inhibitor, but was later discontinued due to liver toxicity [84]. Given this history, it is used as a comparison for other drug studies [85], as well as the subject of study for potential chemical derivatives. Intensive 3D-QSAR (quantitative structure-activity relationship) studies, molecular docking analyses, and MD simulations of 60 tacrine-based inhibitors bound to *Tc*AChE have identified key residues involved in binding to be Tyr70 (PAS), Trp84 (CBS), Tyr121 (PAS), Trp279 (PAS), and Phe330 (CBS) across various binding sites [86].

Tacrine derivatives have been investigated using docking calculations. In a survey of racemic tacrine derivatives in complex with AChE, Maalej et al. found the derivative 4-(13-amino-10,11,12,14-tetrahydro-9H-benzo[5,6]chromeno[2,3-b]quinolin-14-yl)phenol to be four times more effective in inhibitory activity than tacrine [87]. Tacrine-carbazole hybrids in complex with both AChE and BChE were also investigated via docking by Thiratmatrakul et al., who not only found these hybrids to exhibit a preference for BChE, but also showed them to have a potential for ABTS radical scavenging activity [88]. In the early 2000s, in situ click-chemistry led to the discovery of potent, femtomolar range tacrine derivatives [89], which have since been studied in complex with mouse, *Drosophila melanogaster*, and *Tc*AChE through MD simulations [90]. More recently, the Richardson laboratory employed docking and quantum characterization, which revealed that tacrine derivatives with spacers, such as pentylaminopropene and pentylaminopropane, can increase inhibitor specificity to target BChE [91].

As expected, MD simulations can provide more detailed insight into tacrine-cholinesterase complexes, especially when coupled with experimental observations. For example, MD simulations allowed Decker and coworkers to explore interactions between ring-opened and ring-closed cyclohexen-like rings of tacrine derivatives [92], as well as to provide IC_50_ values for indole-3-acetic acid (IAA)-tacrine dual AChE/BChE inhibitors [93]. Other studies using only computational methodologies have focused on tacrine-cholinesterase complex dynamics. While recent QM/MM simulations of tacrine bound to AChE reported by Nascimento et al. demonstrated that van der Waals forces play just as important a role as electrostatics in binding and stabilization of the complex [94], MD simulations of BChE in complex with tacrine reported by Wan et al. emphasize the importance of protonating Glu197 near the catalytic triad, which stabilizes participation of a localized water molecule and leads to preservation of the His438 residue [95].

Interestingly, MD simulations of a survey of tacrine-huprine heterodimers revealed that derivatives show potential for AD and prion disease treatment because they both inhibit the PAS and CAS in AChE while reducing β-amyloid and prion peptide aggregation [96]. Other dimers that exhibit dual-site inhibitory activity with the cholinesterase enzymes are the bis(7)tacrine derivative with S-allylcysteine and cystamine, both of which can also serve as antioxidants [97,98]. The hybrid of tacrine and quercetin, dubbed Tac-Quer in a 2019 study by Habibpour et al., allows tacrine to act as an inhibitor to either AChE or BChE, while quercetin, a well-known metal scavenger, seeks Zn^2+^, Cu^2+^, and Fe^2+^ cations in trace quantities in brain plaque [99].

#### 4.1.2. Galantamine and Derivatives

Galantamine, or (4aS,6R,8aS)-5,6,9,10,11,12-hexahydro-3-methoxy-11-methyl-4aH-[1]benzofuro[3a,3,2-ef][2]benzazepin-6-ol, is another pharmaceutical that targets AD, sold under the brand name Razadyne in the United States [84]. It can be isolated from the *Amaryllidacease* family of plants [100] and, like tacrine, is used as a reference compound in drug discovery [4]. Examination of the X-ray structure of the *Tc*AChE-galantamine complex hinted that galantamine interacts with residue Trp84 at the choline binding site and residues Phe288 and Phe290 at the acyl binding site [38]. Recent docking calculations, however, suggest that galantamine inhibits AChE at the base of the active site gorge [101], and MD simulations point to the importance of hydrogen bonds with water for inhibitor stability in the gorge [102]. While galantamine is larger than the AChE gorge entrance observed in crystal structures, a recent experimental-computational collaboration by Roca et al. illustrated that it can enter the active site pocket of the enzyme following reorientation of the PAS to traffic the ligand inside, as mentioned previously [39].

#### 4.1.3. Donepezil and Derivatives

Donepezil, or 1-benzyl-4-[(5,6-dimethoxy-1-indanon-2-yl)methyl]piperidine, sold under the brand name Aricept [84], also serves as a reference compound in drug discovery studies [102], and was shown in a recent MD study by the Treptow laboratory to act as a mixed competitive and non-competitive inhibitor that interacts strongly with the PAS, ABS, and CAS regions of AChE [103].

QSAR examinations into cholinesterase inhibition have produced handfuls of donepezil derivatives [104,105], indicating that the parent compound is structurally favored to be an inhibitor. Docking simulations of pyridonepezil and quinolinodonepezil derivatives were performed with both cholinesterases, with quinolinodonepezil derivatives proving much less effective at inhibiting human AChE than pyridonepezil derivatives [106]. Marco–Contelles and coworkers took a multi-pronged approach to study donepezil-pyridyl hybrids, which proved to inhibit both cholinesterases at the PAS and CAS, and suggested that the N-alkyl bridge could be used to selectively enhance AChE inhibition by such pyridine-based derivatives [104]. Docking studies by Al-Rashid and Hsung further suggest that the E-ring in the donepezil-like (+)-arisugacin A compound can play a crucial role in binding to AChE [107], and Rahman et al. employed DFT and docking to demonstrate that halogenated derivatives of donepezil, including fluorine and chlorine groups, also show AChE inhibiting potential [108].

In a computational-experimental collaborative study focusing on a series of N-substituted amine derivatives [84], docking and MD simulations were performed using the AChE crystal structure from the pre-formed AChE-donepezil complex and some compounds were observed to mimic the binding pose (position and orientation) of donepezil [109]. In another more recent study, well-tempered metadynamics (WTMtD) simulations of AChE in complex with donepezil by Ghosh et al. showed the protein-ligand complex to increase the ordering of water molecules around Ser203 of the CAS, which discourages ACh from interacting with the active site [110]. Hybrids of donepezil’s benzylpiperidine moiety connected via an oligomethylene linker to an indolyl propargylamino moiety were examined via MD simulations and identified as dual-site binding cholinesterase inhibitors [111]. Docking and MD simulations by Yekta et al. suggest that glycated-AChE (a glycine and AChE complex) poses a challenge for donepezil binding due to the rearrangement of Trp286 and Tyr341 that block this inhibitor from entering the binding cavity [112].

#### 4.1.4. Rivastigmine and Derivatives

Exelon is the brand name for rivastigmine, or [3-[(1*S*)-1-(dimethylamino) ethyl]phenyl] *N*-ethyl-N-methylcarbamate [84]. Docking and MD simulations conducted recently by Ali et al. show that, due to the presence of rivastigmine, TcAChE undergoes carbamylation [102]. Rivastigmine and numerous conformationally restricted analogs were studied by Bolognesi et al. using Monte Carlo calculations, which suggested that the carbamic N-alkyl chain has a more negative effect on binding to AChE than BChE due to the larger acyl binding site present in BChE [113]. Another much more recent study by Wang et al. focusing on a series of chalcone-rivastigmine hybrids support this observation, with results from MD simulations showing rivastigmine hybrids to bind to BChE more easily [114].

#### 4.1.5. Quinazoline and Derivatives

Quinazoline is another pharmaceutical that has been considered as a potential AD treatment. In fact, a number of derivatives have been FDA-approved as anti-cancer and anti-tumor drugs, including Gefitinib, Erlotinib, Vandetanib, Lapatinib, and Afatinib [115]. Although, of the two cholinesterases, quinazoline derivatives seem to bind more effectively to AChE [116], the addition of alicyclic groups to quinazoline analogs increases the binding affinity towards BChE, as these groups bind more effectively to the PAS [117]. Homobivalent quinazolinimes are also derivatives that bind more closely with BChE; here, in docking simulations, the homobivalent quinazolinimes engage with BChE with π-interactions that are absent in the AChE complex [118]. However, in a study by the Decker laboratory tricyclic and tetracyclic quinazoline derivatives led to an “inverted binding mode” with the aliphatic amine in the center [119] and Daoud et al., who used a multi-pronged computational approach to study cholinesterase inhibition, found that pyrazinamide derivatives exhibit strong hydrogen bonding with Tyr121 in *Tc*AChE and Tyr332 in BChE, suggesting that these derivatives are ChE effective inhibitors [120].

#### 4.1.6. Coumarin

Coumarin, or 2H-1-benzopyran-2-one, a natural product found in many plants, has been scrutinized as a potentially potent cholinesterase inhibitor comparable to tacrine. Recent molecular docking simulations by Tanoli et al. reveal that the most potent coumarin derivatives, containing both piperidinyl and ethoxyl groups, lower the binding energy with AChE by nearly 1.5-fold that of tacrine. Large substituents attached to the coumarin ring enable these inhibitors to increase molecular contact with grooves in the enzyme active site gorge including, notably, simultaneous interactions with residues from the choline binding site, the peripheral aromatic site, and the catalytic triad [121]. Coumarin-linked thiourea derivatives exhibit similar binding modes in docking calculations, forming contact with the catalytic triads of both AChE and BChE [122]. However, in this same study, the thiourea group is observed to consistently hydrogen bond with Tyr146 of AChE, with no analogous hydrogen bonding observed for BChE due to the structural differences between the two enzymes. Moreover, hydrophobic interactions appear to dominate electrostatic interactions in these docking results. Another coumarin derivative, 7-hydroxycoumarin, also displays dual binding site capability with both the PAS and CAS of both cholinesterases [123]. A recent docking and MD study conducted by the Mubarek laboratory indicates that while hydrophobic interactions are dominant in stabilizing AChE in complex with coumarin derivatives, structural stability of BChE in complex with these species is predominantly due to hydrogen bonding [124].

#### 4.1.7. Other Pharmaceuticals

A variety of other FDA-approved drugs not intended to treat AD have been examined as possible cholinesterase inhibitors using computational methods. For example, adamantyl-based ester derivatives, which have been more widely used as acne, type 2 diabetes, and anti-viral medications, were studied in complex with both cholinesterases via docking; compounds with a methoxy substituent at position three on the phenyl ring showed the highest potential of binding strongly to both AChE and BChE [125].

Curiously, a number of marine metabolites, which show promising capabilities as pharmaceuticals, were docked with AChE and analyzed as potential treatments for AD [126]. From a database of FDA-approved drugs, Hassan et al. recently employed a screening technique that chose five drugs with higher capacity for AChE inhibition: Risperidone (for schizophrenia and bipolar disorder), Domperidone (for nausea and vomiting), Verapamil (for high blood pressure), Tamsulosin (for enlarged prostate), and Cinitapride (for nausea and ulcers); MD simulations indicated that all complexes were stable [127].

Another recent study by Ozer and coworkers employed docking calculations to understand interactions between BChE and fluoxetine, also known as Prozac, which is commonly used to treat anxiety, obsessive compulsive disorder, and anorexia [128]. Coupled with experimental kinetics measurements, fluoxetine proved to be a competitive inhibitor of BChE that binds deep in the active site gorge [128]. Previous MD and docking studies of BChE in complex with berberine derivatives, compounds found in medications for diabetes and high cholesterol, indicated that Trp82 (CBS), Gly117 (OAH), Trp231 (ABS), and Phe329 (CBS) are all important for binding [129]. Pyridoxine, commonly known as vitamin B6, is also recognized for its AChE inhibition ability: MD simulations of this complex indicate that the ligand forms a covalent bond with Ser203 of the catalytic triad, thus creating a steric barrier for acetylcholine [130].

### 4.2. Narcotics

Another field of inhibition centers around the interactions between narcotics and the cholinesterases, particularly BChE. As mentioned above, BChE is a promiscuous plasma enzyme, and can thus hydrolyze a variety of substrates during circulation. For example, BChE is one of the enzymes responsible for hydrolyzing, and subsequently activating, heroin, making it an ideal enzyme to target as a treatment for heroin overdose. In a recent study by Zhou et al. that addresses the need to block the activation of heroin by BChE, novel inhibitors from solanaceous alkaloid scaffolds were discovered via virtual screening, thus identifying a series of highly selective BChE inhibitors [131].

Recent docking and MD studies have also investigated cholinesterase interactions with nicotine and numerous derivatives thereof. For example, investigations into nicotine-AChE complexes using MD simulations, in tandem with experimental efforts, showed R-nicotine to more strongly disturb the secondary structure of, and to be a stronger inhibitor of, AChE than the S-nicotine analog [132]. Nicotine is the parent compound to the neonicotinoid family, which are present in commercial insecticides and, unsurprisingly, act as agonists to ACh receptors. In a 2018 study, Terali assessed the seven commercially available neonicotinoids using docking calculations, revealing different binding modes with AChE and BChE, and suggesting them to be potential compounds to treat cholinergic and non-cholinergic AD pathogenesis [133].

As discussed in the catalysis section above, cocaine is also a narcotic of interest for cholinesterase studies, and details of the catalytic mechanism of cocaine hydrolysis were discussed in that section. From MD simulations and hydrogen bonding energy (HBE) calculations, the energy barrier for hydrolysis of ACh by each enzyme was compared to the hydrolysis of (+)- and (−)-cocaine by human BChE and both of these differences were found to be approximately 3–5 kcal/mol [62]. This energy difference is attributed to the fact that only Gly117 and Ala199 in the OAH of BChE interacts with the carbonyl oxygen of cocaine, with Gly116 not participating [62,134].

A focal point in computational research of BChE-cocaine complexes is how BChE can be mutated to hydrolyze cocaine faster and remain in circulation longer as a possible treatment for cocaine overdose [30]. For example, MD simulations uncovered that mutations of Phe547, Met554, and Phe561 (in the C-terminus section of hBChE) to more hydrophobic residues may increase its circulation [30]. Circulation time of BChE may also be increased due to the introduction of more cross-subunit disulfide bonds, resulting in higher dimer stability as suggested in an MD study by Fang et al. [135].

**Figure 5:**
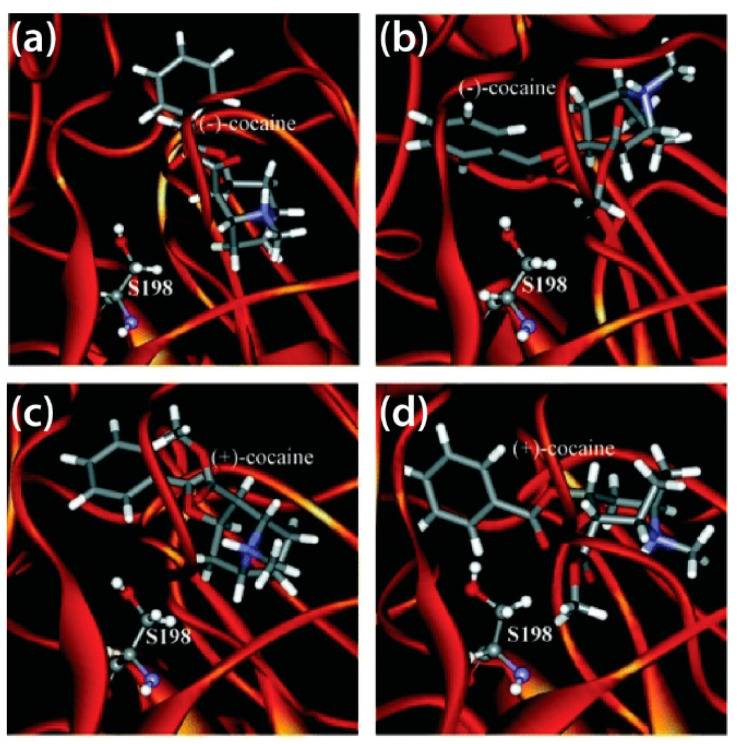
Wild-type BChE bound to (**a**) (−)-cocaine in a non-prereactive complex, (**b**) (−)-cocaine in a prereactive complex, (**c**) (+)-cocaine in a non-prereactive complex, and (**d**) (+)-cocaine prereactive complex [138].

Decreasing the activation energy for transition states, particularly for the rate-determining step, is another approach to amplify BChE catalytic efficiency. After combined computational and experimental studies revealed the rate determining step for the Ala328Trp/Tyr332Ala and Ala328Trp/Tyr332Gly BChE mutants [78,136], MD simulations and virtual screening techniques were employed to determine how these mutations could lower these energy barriers, eventually yielding a mutant that was approximately 2000-fold more catalytically efficient than WT BChE, the 5-point mutant Ala199Ser/Phe227Ala/Ser287Gly/Ala328Trp/Tyr332Gly [137]. The aforementioned Ala328Trp/Tyr332Ala and Ala328Trp/Tyr332Gly BChE mutants were identified as being more catalytically active than WT human BChE, whose binding modes with (−)- and (+)-cocaine isomers as prereactive and non-prereactive complexes are displayed in Figure 5 [138]. The Ala328Trp/Tyr332Ala/Tyr419Ser mutant studied therein lost catalytic potency, as the conformation in which cocaine binds to this mutant is not conducive to catalysis [138]. Not all BChE mutants, however, are more catalytically efficient than WT BChE. Prompted by kinetics studies [139,140], a 2015 MD study of mouse and human BChE, alongside their Ala199Ser/Ser227Ala/Ser287Gly/Ala328Trp/Tyr332Gly mutants, showed that the parent enzymes are approximately 250-fold more catalytically effective than their derivative hydrolases [141].

### 4.3. Organophosphates

#### 4.3.1. Reversible Inhibition

Many organophosphates (OPs) with good leaving groups (weak bases) bind irreversibly to both cholinesterases, and molecular recognition complexes have been examined with docking and MD simulations. Human, mouse, and housefly models of AChE, as well as horse BChE, in complex with O,O-dialkylphosphate inhibitors suggest that the amino acid at position 400, which is either valine or phenylalanine, plays a key role in determining how well the OP will bind in the pocket based on the bulkiness of that amino acid [142]. Furthermore, Lee and Barron studied insect and mouse AChE with OP inhibitors using docking/QSAR techniques, demonstrating that Leu328 in the acyl binding site of insect AChE allows for less enzyme specificity in comparison to Phe295 of mouse AChE, and emphasized the importance of-interactions with the OAH [6].

Around this same time, Veselinovic et al. used Monte Carlo as part of their QSAR analysis to identify the best AChE inhibitors from a database of 278 OP compounds, with the goal of reducing cholinergic activity [143]. In recent months, Yang et al. published their combined Monte Carlo/MD study of *Tc*AChE adsorption in charged monolayers, which revealed that binding sites in the active site gorge orient themselves toward positively charged surfaces and away from negatively charged surfaces, a somewhat intuitive result given that cholinesterases have evolved to attract and hydrolyze positively charged choline moieties, but also providing useful insight for experimentalists using AChE as a means to detect OP compounds [144]. Also reported in the last few months were docking calculations of *Electrophorus electricus* AChE in complex with the voluminous and negatively charged 12-tungstosilicic acid and 12-tungstophosphoric acid, which allowed for detection of a previously-unknown allosteric binding site that has been subsequently labeled β-AS [145].

BChE-OP molecular recognition complexes have also been investigated by the Sorin laboratory using docking and MD methods. In a collaborative 2017 study that featured experimental work, the structural basis for relative *K*_I_ values was probed via massive docking calculations for an assortment of dialkyl and aryl phosphate inhibitors in complex with BChE [146]. That same year, a massively-parallel MD study involving a very limited number of dialkyl phenyl phosphates probed the entropy change associated with binding to these OPs, and demonstrated there to be residual entropy associated with larger, more complex inhibitors that can sample from a much broader array of binding microstates (poses), thus adding to the stability of those larger and more flexible inhibitors entropically [147].

**Figure 6:**
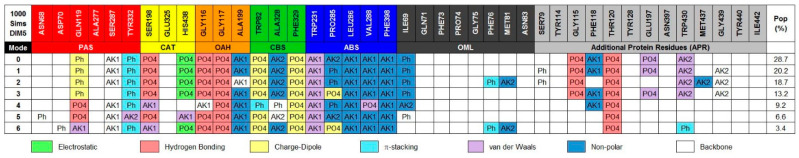
Contact table for the DIM5 inhibitor in BChE binding pocket. SWIFs were taken post 80 ns from one thousand 110 ns MD simulations [148].

More recently, this same laboratory studied an array of dialkyl phenyl phosphate inhibitors via MD simulation, including numerous phenyl substitutions that had been previously probed via docking calculations [146] and a small set of alkyl-to-cholinyl substitutions to mimic the chemistry of the natural substrates. It was noted therein that larger R-groups increase van der Waals contact area between the enzyme and the ligand, with S-enantiomers apparently binding more strongly than their R analogs [35]. In an effort to characterize the observed modes of binding in the flexible BChE-OP complexes, contact tables such as that shown in Figure 6 were used to highlight specific interactions between portions of the inhibitor and specific binding sites and amino acids in the BChE active site gorge. Each row below the label rows at the top represents a binding mode, and every column is an amino acid residue known to participate in binding. Here, contacts are identified as chemical groups separated by 5 Å or less and the cell entries report which functional group dominates that interaction based on relative intermolecular interaction strength.

In a 2020 follow-up study, these BChE-OP were subject to massively-parallel MD simulation and then used as model systems around which to develop a methodology for accurately identifying binding modes from such rich data sets [148]. The resulting technique, brute force *k*-means clustering of surface-weighted interaction fingerprints (SWIFs), employs simple and intuitive statistical criteria to identify binding modes, and bypasses the heuristic nature of the *k*-means clustering algorithm. The contact table in Figure 6, taken from this most recent publication [148], demonstrates distinct binding modes for the OP inhibitor that binds most strongly to BChE of those so far studied by that laboratory and their collaborators, with *K*_I_ = 1(±0.4) μM.

#### 4.3.2. Irreversible Inhibition, Activation, and Reactivation

While the section above centered on reversible molecular recognition of OP inhibitors, “aged” cholinesterases are those that have undergone phosphorylation and have experienced significant structural change, thereby rendering them catalytically impotent. A survey of the energy landscape of the acyl pocket loop uncovered that the products of the reaction between AChE and diisopropyl fluorophosphate deviate significantly from the AChE crystal structure [149].

Like some narcotic and catalysis studies, researchers have focused on how BChE mutants can address and add insight to OP poisoning. For instance, Dwyer et al. found that BChE mutants Tyr332Ser, Asp340His, and Tyr332Ser/Asp340His all resist nerve agents by modifying the size of the “main door” [150]. However, in the case that the enzyme is already bound to the OP, Masson et al. focused on transition states in order to find more catalytically efficient mutants [151], and a more recent study by this same team showed that other mutations, such as Asn322Glu/Glu325Gly with an alternate Ser198, His438, Asn322Glu catalytic triad, allow complexes to self-reactivate [60].

**Figure 7:**
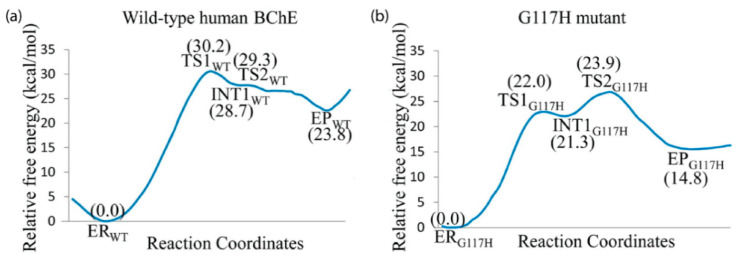
Free energy profiles for the reactivation of sarin-phosphorylated human BChE for (**a**) wild type hBChE and (**b**) the G117H mutant discussed in the text [155].

MD simulations have also shown that minor mutations, such as replacing Gly116 in the OAH of BChE, could cause severe structural deviations [152], and simulations of the Gly117His and Gly117Asp mutants of BChE revealed the presence of a water molecule near Ser198 in the catalytic triad, which may be responsible for the reordering of water and subsequent conformational changes present in the mutants [152]. When these water molecules are replaced by glycerol molecules in cresyl saligenin phosphate-phosphorylated BChE, there is a conformational change caused by His438 leading to a less reactive intermediate complex [5]. The structure of the Gly117His BChE mutant, studied via X-ray by Nachon et al. [153], was probed by Amitay and Shurki, who identified a single conformation from a set of computationally-generated structures that would fully reproduce the acetylation of acetylthiocholine [154]. The Gly117His BChE mutant was further explored as a potential OP bioscavenger in a QM/MM study by Yao et al. that reported improved activity against sarin by reducing the rate-determining energy barrier compared to wild type hBChE [155], as demonstrated in Figure 7.

The introduction of another compound as a reactivator to aged, or phosphorylated, cholinesterase has also been considered. Many reactivation studies have centered around AChE because of its crucial role in neurotransmission, which is interrupted by the introduction of nerve agents and insecticides, but a recent structure-based study suggested that small molecules (<200 Da), such as oximes, may act as strong antidotes for OP-poisoned AChE [9]. Oximes have been widely explored as reactivators for deactivated cholinesterases, so it is no surprise that the structure-activity relationship and docking studies proposed oxime-based compounds as antidotes for cholinesterases poisoned by OPs [156,157].

Nerve agents such as VX (venomous agent) and sarin (GB, as classified by the US-American military) have also been a focal point of studies on cholinesterase reactivation. Oximes used as reactivating compounds for OP-poisoned AChE are supported by computational and experimental research [158]. The prereactive complex of the oxime HI-6 and *Mus musculus* AChE covalently inhibited by sarin was examined by Allgardson et al., whose X-ray investigations and DFT calculations provided an essential foundation for research into the reactivation mechanism of OP-poisoned AChE [159]. Monte Carlo calculations by Veselinovic et al. of AChE-sarin reactivation reiterated that pyridinium oximes are decent antidotes [160]. A more recent study of charged and uncharged oximes by de Souza et al. compared these species with VX- and GB-poisoned AChE: while charged oximes proved to outperform the uncharged oximes, it is also an unfortunate reality that charged oximes do not cross the blood-brain barrier very well, making physical intake of the better reactivator more difficult [161]. Despite this setback, oximes are generally explored in more depth compared to pre-exposure antidote carbamates because carbamates also change the AChE structure via carbamylation [162].

Tabun (GA, as designated by the US-American military) is, unlike other nerve agents, particularly resistant to oxime compounds as reactivators [163]. This resistant quality has motivated researchers to find more effective oxime derivatives for tabun-cholinesterase complex reactivators. Dimethyl(pyridin-2-yl)sulfonium based oximes were examined at the DFT M05-2X/6-31G* level and determined to be better reactivators, as they lower the energy barrier by 4.4 kcal/mol [164], and hierarchical ab initio calculations revealed that charged oxime derivatives as antidotes to tabun bound AChE are stronger than normal oxime compounds due to specific stereoelectronic characteristics [163]. Indeed, a 2014 study by Lo and Ganguly found charged oximes to be more effective than their uncharged analogs, and their QM/MM studies further suggested that N-(pyridin-2-yl)hydroxylamine is a better antidote than traditional oxime treatments and that it has a similar IC_50_ value [165].

Treatments for general nerve agent and insecticide poisoning have utilized oxime derivatives as well. Reactivation of a VX-AChE complex using a deprotonated pralidoxime, or 2-pralidoxime (2-PAM), occurs through consecutive addition-elimination steps and shows promising results as an antidote [166]. Docking and QM/MM methods paired with experimental observations revealed that trimedoximes show potential to reactivate *Mus musculus* AChE, with the AChE-VX complex showing the best results [167], and MD simulations of 2-PAM with phosphorylated AChE support this claim [168]. The importance of protonated Glu202 in the reactivation of VX-inhibited mouse AChE was observed in QM/MM simulations performed by Driant et al. [169]. Further, symmetrical and unsymmetrical isoquinolinium-5-carbaldoximes showed strong inhibition for both cholinesterases; the weaker inhibitors were selected for additional experimental and computational investigation [170]. Interestingly, QSAR studies found that a combination compound consisting of tacrine and aroylacrylic acid phenylamide moieties showed potential as pre-exposure OP-poisoning antidotes [171].

### 4.4. Other Organic Moieties

#### 4.4.1. Hydrocarbons

The Sepčić laboratory studied the interactions of the carbon-based nanomaterials (NM) carbon black (CB), fullerene (C_60_), and graphene oxide (GO) in complex with AChE experimentally and with docking and MD simulations, finding that CB inhibited AChE most efficiently, while C_60_ was least efficient and interactions with the GO surface allowed AChE to retain its native shape and activity [172].

Flavonoids are targeted as potential inhibitors that are not regulated by the FDA. Vats et al. found a number of flavonoid analogues to be novel AChE inhibitors via QSAR analysis [173]. Another sub-class of flavonoids are catechins, including hydroxyl-rich epicatechin, which has been undergoing trials as a potential therapeutic for diabetes and cancer. Of these, epicatechin 3,5-O-digallate was investigated with docking and MD simulations in complex with BChE and found to bind closely to the His484 residue of the catalytic triad with as many as six stabilizing hydrogen bonds [174].

As noted above, investigations into cholinesterase structure and function in the presence of certain toxins are of significant interest. The behavior of aflatoxin, for example, which is regulated by the FDA, not as a pharmaceutical but as a toxin, was examined by Sanson et al. in complex with AChE using MD simulations [50], which revealed that the presence of aflatoxin and its interaction with Trp84 (in the CBS) caused enlargement of the active site gorge [50]. Furthermore, a recent study by the de Almeida laboratory showed aflatoxin M1, a toxic natural compound found in contaminated dairy products, to be another potential inhibitor of AChE, which binds to the CAS region of AChE but does not bind to or inhibitor BChE [175].

Other compounds that have been considered as BChE inhibitors include derivatives of 2-phenylbenzofuran [176]. MD simulations revealed that, while these derivatives bound to the PAS and CAS sites quite well, a derivative that included a para-position hydroxy group on the phenyl moiety improved inhibition against BChE [176]. Phenyl valerate is another aromatic hydrocarbon that has been studied recently in complex with BChE by Estevez et al.: they observed via MD simulation that phenyl valerate inhibits BChE at different ends of the active site gorge, thereby inhibiting the hydrolysis of ACh; it was experimentally determined, however, that both phenyl valerate and ACh can be hydrolyzed simultaneously [177], highlighting the need for extensive simulation time with respect to complex systems and caution when interpreting the results of those simulations.

#### 4.4.2. Nitrogenous Compounds

##### Amines

Amines are one of the most common functional groups occurring in nature. As a reminder, compounds are placed in this section due to the general abundance or consistent occurrence of amine groups in the inhibitor series studied in a given paper. For example, piperidine is an amine-containing compound that has been considered as a cholinesterase inhibitor in a handful of publications, including phenoxyethyl piperidine derivatives, which were studied via MD simulation in complex with electric eel AChE and horse BChE [178]. The phenoxyethyl derivatives most structurally similar to donepezil had the ability to bind to both the CAS and PAS, while many others bound only to the CAS [178]. In contrast, piperidine compounds substituted with arylaminopropanone were recently examined by Hudcova et al. via docking, MD, and QM/MM approaches and were compared to rivastigmine and galantamine [179]. In fact, our current appreciation for the role of hydrophobic active sites residues in binding is highlighted by a previous study by Khayamian and coworkers, who tested 68 piperidine and amine compounds as AChE inhibitors using docking and MD simulations, thereby confirming that hydrophobic interactions are a dominant factor in cholinesterase-inhibitor binding [180].

A handful of other amine compounds have been considered as possible cholinesterase inhibitors. Docking simulations of 4-acetoxy-plakinamine B in complex with AChE uncovered that the inhibitor binds primarily with the PAS and ABS [181]. Moreover, Shrivastava et al. recently studied 23 p-aminobenzoic acid derivatives in complex with both AChE and BChE, which were compared to the binding affinity of donepezil [182], and 1H-benzimidazole compounds with amine substituents were shown, in a previous study, to prefer BChE in docking and MD simulations [183]. Indeed, around 85 amine-containing compounds were used as an input to a QSAR study by Abuhamdah et al., with 24 compounds exhibiting micromolar IC_50_ values [184].

##### Amides, Imides, Imines, and Carbamates

Other miscellaneous but common nitrogenous functional groups and compound types are amides, imines, and carbamates. Imides are one of the least examined groups, which is acceptable given the large swath of papers with inhibitor series that have little in common with each other. A QSAR analysis of 84 N-aryl-monosubstituted derivates provided 42 imide inhibitors and emphasized the importance of-interactions with the Trp82 and Trp86 residues in BChE and AChE, respectively, via MD simulations [185]. Imines are close behind, with N-(1-(5-bromo-2-hydroxyphenyl)-ethylidene)-3,4,5-trihydroxybenzohydrazide, a Schiff base derivative and the most potent AChE inhibitor in this series of compounds, revealed via docking calculations to interact mostly with the PAS and ABS [186], as was reported above.

Amide compounds have been more widely studied as cholinesterase inhibitors, including anandamides and acylethanolamides (NAEs), commonly found in most tissues, along with oleoylethanolamide and palmitoylethanolamide, which were docked with BChE; the latter were found to be uncompetitive inhibitors of BChE, and anandamides were found to be noncompetitive [187]. Docking and MD were also utilized to study the inhibitory activity of 4-aryl-oxo-2-aminylbutamides with both AChE and BChE. Although many of these compounds were ineffective towards AChE, the most potent AChE inhibitors displayed a tendency for stronger interactions between -NH moiety and the Tyr124 hydroxyl in the PAS [188]. More recently, Singh and Gupta used a multi-pronged QSAR analysis, docking, and MD approach to study a series of potential AChE inhibitors in which over half contained amide groups, reporting that inhibitors lacking amide groups received lower docking scores [189].

Carbamates are structurally similar to amides and carbamate-based inhibitors thus behave similarly to amide-based inhibitors. Recently reported RMSD calculations of carbamate-based inhibitors in complex with AChE were found to reach structural equilibrium after about 6 ns of MD simulation time and demonstrated that carbamate inhibitors with aromatic rings were more strongly drawn to AChE’s binding pocket [190]. Indeed, from another recent MD-based study of thymol carbamates in complex with BChE, the importance of hydrophobic interactions with Trp82 (CBS), Gly116 (OAH), and Gly197 was emphasized in relation to MR-complex stability, as were water mediated interactions within the complex [191]. Analogously, the stability of AChE-inhibitor complexes was found to depend strongly on hydrophobic interactions with Tyr341 and Trp286 (near gorge entrance) and hydrogen bonds with Tyr124 (also near gorge entrance) and Phe295 (farther down inside the gorge) [191].

##### Nitrogenous Heterocyclic Rings and Derivatives

One class of molecule common to this group are indoles and indole derivatives, a number of which have been studied via docking. For example, docking calculations by Dominguez et al. with AChE revealed that meta-substituted benzylamine indole derivatives outperformed other indole cholinesterase inhibitors [192]. Dileep et al. also docked indole-3-acetic acid (IAA) and indole 3-butyric acid (IBA) derivatives with AChE; these indoles are known as auxins, or plant growth regulators, which are used in culture experiments for plant tissue [193]. Most recently, Bingul et al. docked six 4,6-dimethoxyindole based hydrazide-hydrazones that were found to bind to both AChE and BChE more strongly if they contained a phenyl group [194].

Piperine, a chemical closely associated with black pepper, has also been investigated as a potential cholinesterase inhibitor. Arylaminopropanone derivatives substituted with piperidine were docked and simulated with both cholinesterases, proving that they perform most similarly to galantamine and rivastigmine among the series of arylaminopropanone with N-phenylcarbamate moieties [179]. In another recent docking study, piperidine and curcumin, a chemical found in turmeric plants, were found to bind most closely to AChE, with binding energies of −10.5 kcal/mol and −9.6 kcal/mol, respectively [195], and it has been shown that cholinesterase binding sites most responsible for strong interactions with piperidine, as with many of the species discussed above, are the CAS and the PAS regions of the gorge [196,197].

Docking and MD simulations revealed that isoalloxazine derivatives with an ortho dimethoxybenzyl group were favorably bound to the peripheral binding site of AChE [198]. AChE-docked 4-aminopyridine semicarbazone derivatives with biphenyl rings had stronger hydrophobic interactions and overall better binding affinities than derivatives without those groups [199]. Carbazole-based stilbene derivatives were also docked with both cholinesterases, as well as the Aβ_1-42_ peptide, and displayed potential as a multitarget inhibitor for AD in a 2020 study by Patel et al. [200].

One interesting potential cholinesterase inhibitor comes from the Chilean *Rhodophilia* (Amaryllidaceae) plant. Docking simulations were performed on *Rhodophilia* compounds with the highest alkaloid compositions, with IC_50_ values also reported from in vitro experiments [201]. The Bohorquez laboratory also studied novel N-allyl/propargyl 4-substituted 1,2,3,4-tetrahydroquinoline derivatives in complex with both AChE and BChE. Their results from docking, MM/GBSA simulations, and experimental work showed a high correlation between the calculated binding free energy and inhibitor activity for both cholinesterase targets [202]. Several years later, this same team studied tetrahydroquinoline(THQ)-isoxazole/isoxazoline compounds, which proved to have similar binding modes to galantamine [203]. Furthermore, tricyclic and tetracyclic nitrogen bridgehead compounds in complex with AChE were investigated with docking and MD simulations as potential inhibitors for AD treatment by the Decker group, with their strongest inhibitor in the tens of nanomolar regime [204].

Interestingly, numerous molecules are studied in tandem with other receptors and enzymes. For example, the Decker group effort noted above also used docking and MD to understand the high affinity of their inhibitors for the human histamine H_3_ receptors [204] and Samadi et al. docked heterocyclic substituted alkyl and cycloalkyl propargyl amines to AChE, BChE, and monoamine oxidases, which are also closely linked to AD [205]. Similarly, small benzimidazole-based molecules were studied as both BChE inhibitors and as human cannabinoid receptor agonists, highlighting the possible future development of a dual-acting therapeutic to treat AD [206].

#### 4.4.3. Organosulfates

It is noteworthy that many studies have used inhibitor series that consistently contain sulfur groups. In some cases, sulfur groups are not the intended focal point; however, since sulfur is a common element in nature, it is deserving of a sub-section. One example is a pair of recent studies by Hassan et al., who studied a series of amide and piperazine sulfonamide derivatives via docking with AChE and BChE [207,208], in which the piperazine sulfonamide derivatives with substituted alkenes proved to be the strongest BChE inhibitors [208]. Another recent example comes from the 2019 study of Yang et al., who looked at a series of inhibitors discovered from structure-based pharmacophore virtual screening and included a number of potential inhibitors containing either thiols or sulfones [209].

Phthalimide-dithiocarbamate hybrids were also recently docked with both cholinesterases, revealing binding modes that are comparable to the FDA-regulated donepezil and rivastigmine pharmaceuticals [210]. In addition, an earlier study showed 7H-thiazolo[3,2-b]-1,2,4-triazin-7-one derivatives with two substituents on the phenyl group closest to the sulfur to show promise of dual CAS and PAS binding and AChE inhibition [211]. Interestingly, a pattern of correlation was found between decreasing fluorescence intensity and increasing binding activity of thioflavin-T with AChE, which was confirmed with docking and MD simulations [212].

Clearly, compounds with thiol or other sulfur-containing groups can be quite effective cholinesterase inhibitors, and early MD simulations revealed that a rivastigmine analog with a sulfur system was 192-fold more efficient at inhibiting cholinesterases reversibly than the rivastigmine parent molecule [113]. MD simulations also demonstrated that the non-competitive substrate acetylthiocholine inhibits both cholinesterases at different ends of the active site; despite the partial competition, experimental work points out that acetylthiocholine and ACh can be hydrolyzed virtually simultaneously [177]. The last compound we will mention here, benzothiazepine, was preferentially bound to BChE due to stronger hydrogen bonding, as observed in MD simulations [11].

### 4.5. Proteins, Nucleic Acids, and Salts

#### 4.5.1. Protein and RNA Binding

Cholinesterase inhibition by small proteins and RNAs has also been studied computationally for a number of species. For instance, fasciculin II is a peptidic three-finger snake toxin and was observed in 5 ns MD simulations to bind to the mouth of the active site gorge in mouse AChE, effectively blocking any substrate from entering the gorge [213], as was observed in an X-ray structure from the Sussman laboratory just a few years earlier [214]. AChE was also modeled with cytochrome c (Cyt c), a hemeprotein generally associated with respiratory cell functions, and it was reported that AChE interactions with Cyt c play a crucial role in apoptosome formation. Macro-modeling studies reveal that Cyt c binds to the PAS of AChE, blocking gorge access a la fasciculin, and also that binding modes with AChE are similar regardless of whether the heme group in Cyt c is present (Holo) or absent (Apo) [215].

Sohail and Rashid used docking and MD simulation to study interactions between the RNA recognition motif (RRM), the most abundant RNA-binding protein domain, and BChE. While it is unclear the degree to which RRM-bound BChE would be catalytically efficient, the authors note that gaining a better understanding of these interactions in vivo could prove highly useful in therapeutic development [216]. A recent docking-based follow-up to that article reports the use of microRNA (miR-132) as a potential inhibitor of AChE, with miR-132 binding predominantly via interactions with the catalytic triad, and thus blocking access to the substrate [217].

#### 4.5.2. Nucleobase Derivatives

Most nucleobase derivatives have focused on pyrimidine, a six-membered heterocyclic compound found in DNA and RNA. Examples of these derivatives include a series of di-phenylpyrimidine derivatives that were recently investigated as AChE inhibitors with docking and MD simulations [218,219]. The molecule labeled VB8 by Kumar et al. showed the highest activity against AChE by demonstrating additional (substituent-induced) interactions with the active site gorge [219]. Another recent docking and MD study reveals that uracil derivatives can inhibit both cholinesterases [220], and 6-methyluracil has also been recently modeled in complex with AChE and BChE, showing stronger binding than donepezil and stabilizing secondary binding to the PAS [7,221].

#### 4.5.3. Ion and Salt Binding

Although not all salts are organic, it would be irresponsible to exclude them from this review. A 2020 docking study by Yigit et al. found amine-tethered benzimidazolium salts with a trimethyl benzyl ring to be efficient AChE inhibitors due to the close proximity and strong interaction with the CAS and the PAS [222]. Previously, 2-*N*,*N*-dimethylaminecyclohexyl 1-*N*′,*N*′-dimethylcarbamate isomers and their methylsulfate salts were investigated computationally, in tandem with experimental work, as cholinesterase inhibitors, revealing that the lowest binding rate was 55% and the highest binding rate was 90% with BChE [223].

## 5. Virtual Screening

After exploring the multitudes of cholinesterase inhibitors that have been studied via computation, it is important to recognize progress and state-of-the-art improvements that have been made in the area of virtual screening, particularly as developed for or demonstrated on AChE and BChE. For those not familiar with the term, virtual screening is a computational method by which large libraries of small molecules can be searched for potential matches to a specific drug target. Many virtual screening methods that have been developed specifically in relation to the cholinesterase enzymes. As has been highlighted in numerous sections above, virtual screening studies are typically QSAR studies paired with docking and/or MD simulation; such studies are designed to find chemical entities and assess their potential inhibitory effectiveness [224].

As an example, Discovery Studio 2.5.5. was used to construct pharmacophore models with the goal of finding molecules that inhibit AChE and protect it from amyloid beta toxicity: from a sample of 62 compounds, only nine were found to interact favorably with AChE [225]. Some years later, the same research team used virtual screening to identify BChE-specific inhibitors from commercial databases of comprising 3.9 million compounds: virtual screening, docking, and bioassay reduced the list of possible matches to just six compounds [226]. Similar results have been reported for other virtual screening studies, including a computer-aided workflow that utilized hierarchical, structure-based screening, thereby yielding five potential cholinesterase inhibitors [227]. Furthermore, six inhibitors, three each for AChE and BChE, out of four commercial compound databases were found using structure-based pharmacophore modeling intended for AD treatment [209]. Similarly, both ZINC (zinc15.docking.org) and DrugBank (go.drugbank.com) were screened for reactivation oxime compounds [228]. As a true success story, the Gobec group developed a successful virtual screening method for BChE [229] that recently helped to realize the discovery of some of the most powerful reversible inhibitors of BChE known, with inhibitions constants in the picomolar to nanomolar range [230].

There are indications, however, that advancements in virtual screening remain to be made. For example, an early report of machine learning as a guide to virtual screening produced cholinesterase inhibitors with a wide range of IC_50_ values, suggesting that certain proposed inhibitors may be too toxic [231]. Detecting false positives would also improve virtual screening studies, an issue that was addressed when screening compounds from Maybridge.com and ChemBridge.com databases for potential BChE inhibitors, in which five ligands were chosen after ADMET calculations, Lipinski’s Rule of Five, and docking simulations [232]. Accounting for inhibitor stereochemistry with respect to active site geometry is another factor to be addressed. For example, virtual screening studies of 24 chiral organophosphates revealed that S-isomers exhibited stronger inhibitory activity towards AChE than their respective R-isomers [233].

Given their pronounced gorges and well-defined binding sites, along with the extensive literature on cholinesterase studies, it is no surprise that AChE and BChE have been used as example enzymes in a number of virtual screening studies. Some methods take hands-on approaches, such as utilization of a Monte Carlo approach paired with CORAL calculations [234]. Employment of steered MD simulations that calculate the work needed to remove the ligand from the binding site proved to be more efficient than conventional MD simulations and subsequent calculations of binding energies [235]. Using docking simulations as a means of assessing ligand mobility, a factor that is considered in virtual screening, was also demonstrated on BChE [236]. Automated docking software, such as ICM-Pro [146] and AutoDock [237], has also proven insightful in recent studies.

In contrast, other researchers have developed their own virtual screening algorithms to focus solely on the cholinesterases. For example, SHAFTS (SHApe FeaTure Similarity) is a 3D similarity calculation designed for AChE ligand discovery [238] and LiSiCa (Ligand Similarity using Clique algorithm) is a virtual screening development featuring BChE [239]. Lastly, the ADAM&EVE virtual screening method was presented with a focus on AChE inhibition and identified thirteen potential compounds from an original database of 160,000 [240]. It will indeed be exciting to see the directions in which this area grows in the years to come.

## 6. Conclusions

Cholinesterase structure, function, and inhibition have proven to be a source of great interest for the application of a broad spectrum of modeling and computational approaches. This review examined articles that detailed the mechanisms by which substrates and inhibitors locate and enter the gorges of AChE and BChE, how specific binding sites within the active site gorge of these enzymes respond to and interact with specific gorge binding sites, and pathways that the products of hydrolysis and other small molecules may find to enter or leave the active site. Also reviewed were computational studies of the mechanisms and thermodynamics of substrate hydrolysis by both enzymes, revealing that AChE and BChE have distinct rate-determining steps. Catalysis studies directed at BChE hydrolysis of cocaine, and the various mutations that can speed up the catalytic process, were also explored in depth.

The bulk of our findings, however, revolved around inhibition. Some major pharmaceutical compounds such as tacrine, galantamine, donepezil, rivastigmine, quinazoline, and coumarin—most of which are conventional, well-known medications for the treatment of AD and other human ailments—and their derivatives, were docked or simulated with AChE and BChE. A sizeable group of additional FDA-regulated compounds were also discussed, with the narcotic section highlighting findings regarding the cholinesterases either bound to or hydrolyzing heroin, nicotine, and cocaine. Organophosphates were heavily explored above, including: discussion of various OP compounds as potential reversible inhibitors; the irreversible binding of nerve agents such as sarin, tabun, and VX; and enzyme reactivation by molecules such as oxime derivatives. The final inhibition section, and by far the most diverse, included other organic compounds including various hydrocarbons, and nitrogenous compounds such as amines, amides, carbamates, and nitrogen heterocycles. Other organic molecules discussed were organosulfates, protein and nucleic acid derivatives, and ionic inhibitors.

Finally, advancements in virtual screening methodologies and software were discussed, with some of these methodologies specific to cholinesterases and others simply featuring them as prime applications. Although there have been hundreds of computational cholinesterase studies published in the last two decades, it is exciting to consider the many possible directions that computation will lend itself to improve our understanding of these enzymes and their function in the future, with potential real-world applications in human disease therapies, treatments for pesticide and nerve agent poisoning, and management of drug overdoses.
